# A dataset of interactions and emotions for website user experience evaluation

**DOI:** 10.1038/s41597-025-06079-1

**Published:** 2025-11-17

**Authors:** Andrea Esposito, Giuseppe Desolda, Rosa Lanzilotti

**Affiliations:** Department of Computer Science, University of Bari Aldo Moro, Via E. Orabona 4, 70125 Bari, Italy

**Keywords:** Computer science, Human behaviour

## Abstract

This data descriptor introduces a dataset designed for affective computing applications in the context of human-computer interaction, with a particular focus on user experience (UX) and website interactions. The dataset comprises interaction logs, including mouse movements and key presses, collected over a period of 30 days from various websites. Each recorded interaction is paired with corresponding emotional data, which is encoded according to Ekman’s emotion model, allowing for a nuanced analysis of emotional responses. This dataset is particularly valuable for examining how emotions influence user behaviour, and vice versa, also across different types of websites. Its primary aim is to facilitate the development of Artificial Intelligence (AI) systems capable of detecting user emotions solely based on user interaction data. Such systems have potential applications in improving UX design, personalizing web content, and enhancing the overall UX by adapting to emotional states without requiring explicit input from users.

## Background & Summary

User eXperience (UX) has become a critical factor in the success of software products, especially in the domain of websites. Over the past few decades, researchers and practitioners have recognized UX as a multifaceted concept that goes beyond the traditional quality of usability. Indeed, while usability emphasizes attributes like ease of learning and ease of use, UX encompasses a broader range of factors, such as emotional engagement and satisfaction derived from the interaction with a system^1–3^. The ISO 9241-11 standard defines UX as “*a person’s perceptions and responses resulting from the use and/or anticipated use of a product, system, or service*”^2^. This definition suggests that UX encompasses not only the functional aspects of a system but also elements related to user emotions, such as pleasure, enjoyment, and even aesthetic appreciation. When designing for UX, the focus expands to include qualities like beauty, emotional resonance, sensory appeal (e.g., tactile sensations), and rhythm^3^.

Despite the growing awareness of UX’s importance, it is frequently overlooked during the software development process^4^. One barrier is the perception that UX evaluation requires significant resources, both in terms of time and specialized expertise, and lacks solutions to automate its evaluation^4–6^. Despite researchers’ attempts, like introducing “discount usability”^7^, traditional usability testing methods, such as in-person user studies, tend to be still perceived as time-consuming and labour-intensive, limiting their accessibility to many development teams. To address this gap, research has increasingly explored the potential of (semi-)automated UX evaluation tools, which could streamline the evaluation process and make it more feasible for teams with limited resources. Though remote testing methods have existed since the mid-1990s and numerous tools are available today^8,9^, (semi-)automatic tools that evaluate UX are still not widely available and mature^10^.

Given the crucial role emotions play in UX (as proven in its definitions), a promising approach to (semi-)automated UX evaluation could include detecting and analysing user emotions during interactions. For instance, Machine Learning (ML) techniques can be employed to identify negative emotions associated with user interactions, potentially flagging them as indicators of UX issues^11^. Thus, emotion-driven analysis may provide valuable insights into the user’s affective state, enabling the identification of UX bottlenecks that might otherwise go undetected through conventional UX metrics and heuristics alone.

In this study, we aim to contribute to advancing automated UX assessment by developing a comprehensive *in-the-wild* dataset that captures real-world interactions with web-based systems. Our dataset is designed to serve as a resource for UX and AI researchers and development teams, providing data that can be leveraged to train and test ML and AI models capable of detecting emotional responses based on users’ interaction logs, potentially identifying UX issues. By focusing on web-based systems, we aim to provide insights into the UX challenges that users might frequently encounter in their daily lives, hoping for more user-centred, data-driven improvements in web-based UX design.

## Methods

**Fig. 1:**
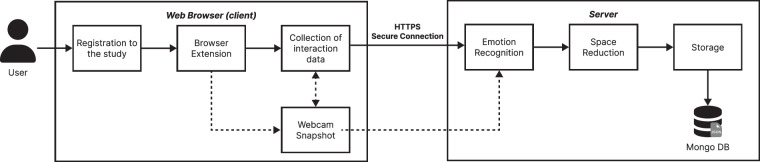
Overall process of the data collection.

We engaged 19 real users (11 males, 8 females, all Caucasian) to develop an in-the-wild dataset capturing real-world human interactions with web applications. All participants volunteered, each providing explicit informed consent through self-registration. No sensitive or identifying information was collected, and all participant-related data included in the dataset is available for analysis. We followed a similar methodology to the one adopted in a previous study^10^. The study was conducted following ethical guidelines, and it received ethical approval from the Ethical Review Board of the University of Bari Aldo Moro (protocol number: 0324775 dated 2024-12-30). Figure 1 provides a summary of the entire data collection workflow.

### Data collection setup

Participants were instructed to install a custom-built browser extension designed for this data collection, which remained active for the one-month duration of the study. Users retained full control over their privacy, with the ability to disable the extension at any time if they chose. To ensure anonymity, each participant was assigned a random identifier that was not linked to any personal information, though basic non-sensitive demographic data was collected when they installed the browser extension. This approach maintains user anonymity while gathering valuable interaction data that might be influenced by the users’ general IT skills and age.

The browser extension captured various features representing the interactions on each web page, such as mouse clicks, scrolling actions, and mouse movements. In addition to interaction tracking, the extension used the participants’ webcams to capture images of their facial expressions at regular intervals of 100 milliseconds. Simultaneously, a snapshot of the users’ interaction state (e.g., page content and actions) is also saved to create a timestamped record of both behavioural and facial data. At 5-second intervals, the accumulated interaction data and facial images are transmitted via HTTPS to a secure web server for further processing. This cycle continued until the user either closed the web browser, switched to another application, or disabled the extension.

### Data processing and storage

Upon reaching the server, the data underwent several processing stages to extract relevant information and minimize storage requirements. The collected webcam images were analysed to infer emotional states using a local version of Affectiva’s AFFDEX SDK^12,13^. This tool employs a state-of-the-art emotion recognition model, leveraging facial expression data to detect Ekman’s seven universal emotions: happiness, sadness, surprise, anger, fear, disgust, and contempt^14^. Following the emotion detection, the extracted emotional values are appended to the interaction dataset, while the original webcam snapshots are immediately deleted permanently to protect user privacy.

While numerous emotion models exist and emotion recognition systems vary in the models they adopt^15,16^, the in-the-wild nature of our data collection required us to constrain our choice. Specifically, we prioritized models that relied solely on facial images, as this approach was more practical and less intrusive for participants. Based on these criteria, we selected Affectiva’s AFFDEX SDK, which has demonstrated reliable performance in previous studies, and its quality is supported by evidence in the academic literature^12,17,18^.

To further optimise storage, the collected features are renamed to only their initials, limiting the verbosity of the dataset (although at the cost of readability). To reduce noise in the emotion data, we applied a filtering criterion based on the output range of Affectiva’s AFFDEX SDK, which returns values between 0 and 100 for most emotions. Only emotion scores greater than 1 were retained for analysis. The only exceptions to this threshold were engagement and valence (the latter ranging from −100 to 100), which were recorded regardless of their value. The final dataset, stored in JSON format, was saved within a MongoDB collection. The final dataset was then reconverted into a flattened CSV for distribution.

### Data aggregation using sliding time windows

**Fig. 2:**
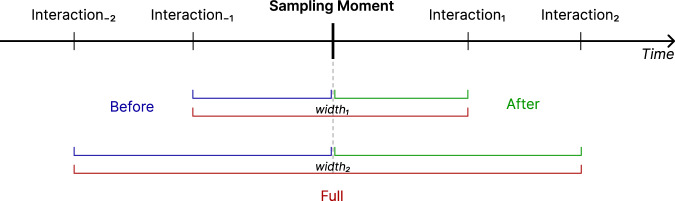
Approach for constructing time windows around emotion sampling moments. Each vertical line represents an interaction log record. Beginning from each sampling moment (centred on the dashed line), multiple time windows are created with the sampling moment as the midpoint. Additional time windows are also generated, using the sampling moment as either the start or end point, capturing interaction patterns both before and after the emotional response. Image adapted from^10^.

The dataset was extended by computing additional features to facilitate and improve analysis. In particular, data was also aggregated into sliding time windows of varying pre-defined widths: 25 ms, 50 ms, 100 ms, 200 ms, 500 ms, 1000 ms, and 2000 ms. Each time window is centred on a record containing emotional data, which is the focal point around which interaction patterns are analysed (see Fig. 2). This structure captures the immediate context around the emotional response, and the progression of user behaviour before and after the emotional event.

For each time window, a range of interaction metrics was computed to provide a comprehensive view of user behaviour. These metrics are better detailed in Section ‘Data Records’. All newly computed features are included in the dataset in an additional file, enhancing its versatility for future analyses.

### Quality assurance and privacy considerations

Strict protocols were implemented throughout the study to uphold participant privacy and ensure data integrity. Each participant was assigned a unique random identifier, effectively minimizing re-identification risks and ensuring that individual identities could not be traced back to the data. Additionally, all data transfers between the browser extension and the server were secured using SSL encryption, protecting data confidentiality during transmission. Importantly, participants retained full control over data collection: they could disable the extension at any time, immediately halting the capture of both interaction logs and webcam snapshots. To further safeguard privacy, the act of disabling the extension was not logged, ensuring participants could opt out without leaving any trace or experiencing external pressure.

To further safeguard privacy, webcam snapshots collected for emotion analysis were automatically deleted from the server immediately after processing. This ensured that facial images were only accessible for the brief period required for emotion detection and were not stored or accessible to researchers at any point. Finally, no personal or sensitive data about the users was collected in the dataset. The keystroke logger was also simplified by recording only the type of key pressed by the users, e.g., letter, number, or special character, to avoid revealing sensitive information.

## Data Records

The dataset collected throughout the study outlined in this data descriptor is fully accessible and freely available on FigShare^19^. It comprises four components: i) demographic information about the participants, ii) details regarding the websites that were visited throughout the collection process, iii) raw interaction data along with the associated emotional responses, and iv) the aggregated interaction data organised into multiple time windows. This section describes the CSV files characterizing the dataset.

**Fig. 3:**
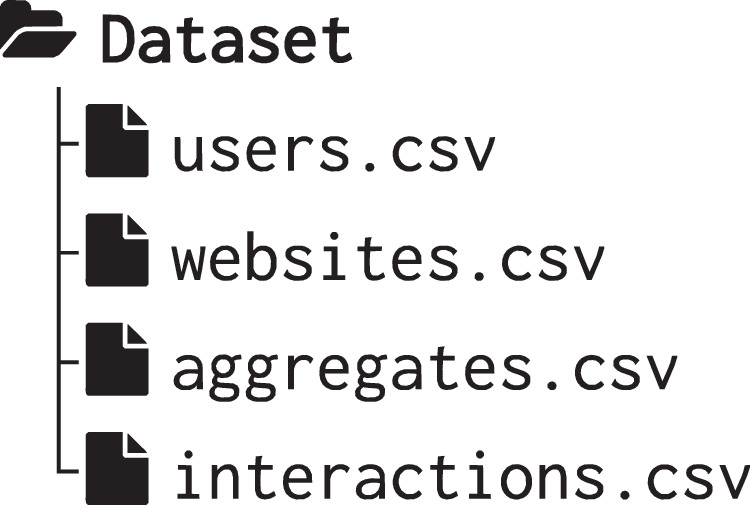
Overview of the dataset structure.

The dataset is structured as a collection of CSV files organized in a hierarchy for easy navigation. At the root level, a file listing participant data (users.csv) and a list of visited websites (websites.csv) are available. Two additional files form the core of the dataset: interactions.csv captures raw interaction data along with emotional responses; aggregate.csv holds time-window-aggregated data for each user. Figure 3 provides an overview of the available data.

The participant’s demographic data includes the following attributes:

- **id** *(String)*: A unique, anonymized identifier represented as a random string of characters;
- **age** *(Integer)*: An integer indicating the participant’s age group, coded as follows: 0 is used to represent under 18 participants; 1 represents the range 18–29; 2 represents the range 30–39; 3 represents the range 40–49; 4 represents the range 50–59; 6 represents participants over 60;
- **internet** *(Integer)*: The average number of hours per day the participant spends on the internet, providing an indication of his/her internet usage habits;
- **gender** *(String)*: The participant’s self-identified gender, recorded as “m” for male, “f” for female, or “a” for non-binary.

The information regarding websites, available in websites.csv, contains information about each website visited throughout the study. Each record in the file includes the following attributes: the URL of the home page of the website (“url”) and an integer indicating the number of interaction records associated with that website (“count”).

All information contained in the interactions.csv file is detailed in the following list, which provides an overview of each attribute collected regarding the raw user interactions. This includes the attribute names, types, and a brief description.

- **id** *(String)*: A unique identifier for the record
- **user_id** *(String)*: The ID of the participant
- **timestamp** *(Integer)*: The timestamp (in milliseconds) in which the record was collected
- **url** *(String)*: The URL the user was visiting at the time of collection
- **mouse.position.x** *(Integer)*: The absolute position of the mouse cursor on the horizontal axis (when 0, the mouse lays on the left edge of the browser viewport, regardless of zoom). This is independent from the current scrolling or zoom factor of the viewport, thus the exact same position may refer to different elements of the website. To understand which website’s element the mouse was hovering, this must be used in conjunction with other features (i.e., absolute or relative scroll).
- **mouse.position.y** *(Integer)*: The absolute position of the mouse cursor on the vertical axis (when 0, the mouse lays on the top edge of the browser viewport, regardless of zoom). This is independent from the current scrolling or zoom factor of the viewport, thus the exact same position may refer to different elements of the website. To understand which website’s element the mouse was hovering, this must be used in conjunction with other features (i.e., absolute or relative scroll).
- **mouse.clicks** *(Boolean)*: Whether at least one mouse button was pressed
- **mouse.clicks.left** *(Boolean)*: Whether the left mouse button was clicked
- **mouse.clicks.right** *(Boolean)*: Whether the right mouse button was clicked
- **mouse.clicks.middle** *(Boolean)*: Whether the middle mouse button was clicked
- **mouse.clicks.others** *(Boolean)*: Whether other mouse buttons were clicked
- **mouse.speed** *(Float)*: The speed of the mouse movement (computed with respect to the last collected instance): supposing that $({x}_{i},\,{y}_{i})$ is the mouse position and ${t}_{i}$ is the timestamp of a given record $i$, ${v}_{i}=\sqrt{{{v}_{x}}_{i}^{2}+{{v}_{y}}_{i}^{2}}=\frac{\sqrt{{\left({x}_{i}-{x}_{i-1}\right)}^{2}+{\left({y}_{i}-{y}_{i-1}\right)}^{2}}}{{t}_{i}-{t}_{i-1}}$.
- **mouse.speed.x** *(Float)*: The speed of the mouse movement on the horizontal axis (computed with respect to the last collected instance): supposing that $({x}_{i},\,{y}_{i})$ is the mouse position and ${t}_{i}$ is the timestamp of a given record $i$, ${{v}_{x}}_{i}=\frac{{x}_{i}-{x}_{i-1}}{{t}_{i}-{t}_{i-1}}$.
- **mouse.speed.y** *(Float)*: The speed of the mouse movement on the vertical axis (computed with respect to the last collected instance): supposing that $({x}_{i},\,{y}_{i})$ is the mouse position and ${t}_{i}$ is the timestamp of a given record $i$, ${{v}_{y}}_{i}=\frac{{y}_{i}-{y}_{i-1}}{{t}_{i}-{t}_{i-1}}$.
- **mouse.acceleration** *(Float)*: The acceleration of the mouse movement (computed with respect to the last collected instance): supposing that ${v}_{i}$ is the mouse speed and ${t}_{i}$ is the timestamp of a given record $i$, ${a}_{i}=\sqrt{{{a}_{x}}_{i}^{2}+{{a}_{y}}_{i}^{2}}=\frac{\sqrt{{\left({{v}_{x}}_{i}-{{v}_{x}}_{i-1}\right)}^{2}+{\left({{v}_{y}}_{i}-{{v}_{y}}_{i-1}\right)}^{2}}}{{t}_{i}-{t}_{i-1}}$.
- **mouse.acceleration.x** *(Float)*: The acceleration of the mouse movement on the horizontal axis (computed with respect to the last collected instance): supposing that ${v}_{{x}_{i}}$ is the mouse speed on the horizontal axis and ${t}_{i}$ is the timestamp of a given record $i$, ${{a}_{x}}_{i}=\frac{{v}_{{x}_{i}}-{v}_{{x}_{i-1}}}{{t}_{i}-{t}_{i-1}}$.
- **mouse.acceleration.y** *(Float)*: The acceleration of the mouse movement on the vertical axis (computed with respect to the last collected instance): supposing that ${v}_{{y}_{i}}$ is the mouse speed on the vertical axis and ${t}_{i}$ is the timestamp of a given record $i$, ${{a}_{y}}_{i}=\frac{{v}_{{y}_{i}}-{{v}_{y}}_{i-1}}{{t}_{i}-{t}_{i-1}}$.
- **trajectory.slope** *(Float [−∞, +∞] or NaN)*: The slope of the mouse trajectory (computed with respect to the previous collected record): supposing that $({x}_{i},\,{y}_{i})$ is the mouse position of a given record $i$, ${m}_{i}=\frac{{y}_{i}-{y}_{i-1}}{{x}_{i}-{x}_{i-1}}$. If the value is $\pm \infty$, the mouse moved vertically. A value of *NaN*, instead, signals that the mouse did not move at all with respect to the previous record (i.e., both the horizontal and vertical speed are zero).
- **scroll.absolute.x** *(Float)*: The $x$ coordinate of the left edge of the current viewport.
- **scroll.absolute.y** *(Float)*: The $y$ coordinate of the top edge of the current viewport.
- **scroll.relative.x** *(Float, [0, 100])*: The relative scroll position on the $x$ axis, obtained by dividing scroll.absolute.x by the total website width.
- **scroll.relative.y** *(Float, [0, 100])*: The relative scroll position on the $y$ axis, obtained by dividing scroll.absolute.y by the total website height.
- **keyboard** *(Boolean)*: Whether a keyboard key was pressed
- **keyboard.alpha** *(Boolean)*: Whether an alphabetic key was pressed
- **keyboard.numeric** *(Boolean)*: Whether a numeric key was pressed
- **keyboard.function** *(Boolean)*: Whether a function key was pressed (e.g., “Alt”, “Control”, etc.)
- **keyboard.symbol** *(Boolean)*: Whether a symbolic key was pressed
- **emotions.automatic_sampling** *(Boolean)*: Whether the record was automatically collected alongside a webcam snapshot. If true, the record *may* contain emotion-related data.
- **emotions.exist** *(Boolean)*: Whether the record is associated to emotional values. This is true if and only if the emotions.automatic_sampling feature is true and a face has been detected in the webcam snapshot.
- **emotions.joy** *(Float, [0, 100])*: The value of the emotion “joy” as detected by Affectiva
- **emotions.fear** *(Float, [0, 100])*: The value of the emotion “fear” as detected by Affectiva
- **emotions.disgust** *(Float, [0, 100])*: The value of the emotion “disgust” as detected by Affectiva
- **emotions.sadness** *(Float, [0, 100])*: The value of the emotion “sadness” as detected by Affectiva
- **emotions.anger** *(Float, [0, 100])*: The value of the emotion “anger” as detected by Affectiva
- **emotions.surprise** *(Float, [0, 100])*: The value of the emotion “surprise” as detected by Affectiva
- **emotions.contempt** *(Float, [0, 100])*: The value of the emotion “contempt” as detected by Affectiva
- **emotions.valence** *(Float, [−100, 100])*: The value of “valence” as detected by Affectiva
- **emotions.engagement** *(Float, [0, 100])*: The value of “engagement” as detected by Affectiva

With respect to all Boolean data in this file, like mouse clicks, each record holds a “true” value if the button is pressed while the record is being collected. We must recall that record collection is triggered by either an event or, in case no event is detected, every 200 ms. For this reason, double clicks trigger four events (mouse down, mouse up, mouse down again, mouse up again), and will thus result in a total of four records collected closely in time (with values for “mouse.clicks.left” following the sequence true-false-true-false). Similarly, supposing the mouse stays still and no additional event is triggered, long mouse clicks will be recorded as a single record (with mouse.clicks.left as “true”), repeated every 200 ms, until the mouse button is lifted (triggering the collection of a record with mouse.clicks.left as “false”).

Finally, aggregate.csv contains aggregated interaction data. For each time window, the emotion sampling point (see Fig. 2) is preserved along with all corresponding data from the interactions.csv file, with each attribute key prefixed by “middle”. This allows for focused analysis around the time-based aggregation point. This aggregation is available across seven time-window sizes (25, 50, 100, 200, 500, 1000, 2000 milliseconds), enabling flexible temporal analyses of user interactions and emotional responses. The aggregate.csv file includes the following attributes (note that “TIME_WINDOW_WIDTH” can be “25”, “50”, “100”, “200”, “500”, “1000” or “2000” milliseconds, while the “SLICE” can be “full”, “before”, and “after”, depending on where the emotion sampling point is in the window—respectively, in the middle, at the end, or at the beginning).

- **middle.[FEATURE]** *(Various)* The value of “FEATURE” for the record that was used to create the time window. The available features are the same as the ones reported previously.
- **[TIME_WINDOW_WIDTH].[SLICE].clicks.all.{sum, avg, std}** *(Integer, Float, Float)*: The total and average (with standard deviation) number of records that reported a mouse button click (regardless of which mouse button) in the time window
- **[TIME_WINDOW_WIDTH].[SLICE].clicks.left.{sum, avg, std}** *(Integer, Float, Float)*: The total and average (with standard deviation) number of records that reported a left mouse button click in the time window
- **[TIME_WINDOW_WIDTH].[SLICE].clicks.middle.{sum, avg, std}** *(Integer, Float, Float)*: The total and average (with standard deviation) number of records that reported a middle mouse button click in the time window
- **[TIME_WINDOW_WIDTH].[SLICE].clicks.other.{sum, avg, std}** *(Integer, Float, Float)*: The total and average (with standard deviation) number of records that reported other mouse button clicks in the time window
- **[TIME_WINDOW_WIDTH].[SLICE].clicks.right.{sum, avg, std}** *(Integer, Float, Float)*: The total and average (with standard deviation) number of records that reported a right mouse button click in the time window
- **[TIME_WINDOW_WIDTH].[SLICE].event_times.{sum, avg, std}** *(Integer, Float, Float)*: The total and average (with standard deviation) time between two events in the time window
- **[TIME_WINDOW_WIDTH].[SLICE].idle.{sum, avg, std}** *(Integer, Float, Float)*: The total and average (with standard deviation) idle time in the time window
- **[TIME_WINDOW_WIDTH].[SLICE].keys.all.{sum, avg, std}** *(Integer, Float, Float)*: The total and average (with standard deviation) number of keyboard key presses in the time window
- **[TIME_WINDOW_WIDTH].[SLICE].keys.alphabetic.{sum, avg, std}** *(Integer, Float, Float)*: The total and average (with standard deviation) number of keyboard alphabetic key presses in the time window
- **[TIME_WINDOW_WIDTH].[SLICE].keys.alphanumeric.{sum, avg, std}** *(Integer, Float, Float)*: The total and average (with standard deviation) number of keyboard alphanumeric key presses in the time window
- **[TIME_WINDOW_WIDTH].[SLICE].keys.function.{sum, avg, std}** *(Integer, Float, Float)*: The total and average (with standard deviation) number of keyboard function key presses in the time window
- **[TIME_WINDOW_WIDTH].[SLICE].keys.numeric.{sum, avg, std}** *(Integer, Float, Float)*: The total and average (with standard deviation) number of keyboard numeric key presses in the time window
- **[TIME_WINDOW_WIDTH].[SLICE].keys.symbol.{sum, avg, std}** *(Int, Float, Float)*: The total and average (with standard deviation) number of keyboard symbolic key presses in the time window
- **[TIME_WINDOW_WIDTH].[SLICE].mouse_movements.rate** *(Float)*: The number of times a mouse movement was recorded in the time window divided by the time window’s width
- **[TIME_WINDOW_WIDTH].[SLICE].mouse_movements.total** *(Integer)*: The number of times a mouse movement was recorded in the time window
- **[TIME_WINDOW_WIDTH].[SLICE].scrolls.rate** *(Float)*: The number of times the web page is scrolled in the time window divided by the time window’s width
- **[TIME_WINDOW_WIDTH].[SLICE].scrolls.total** *(Integer)*: The number of times the web page is scrolled in the time window
- **[TIME_WINDOW_WIDTH].[SLICE].slopes.change_rate** *(Float)*: The number of times the mouse changes directions in the time window divided by the time window’s width
- **[TIME_WINDOW_WIDTH].[SLICE].slopes.changes** *(Integer)*: The number of times the mouse changes directions in the time window, detected if the slope has any change without any threshold.
- **[TIME_WINDOW_WIDTH].[SLICE].urls.change_rate** *(Float)*: The number of times the URL changes in the time window divided by the time window’s width
- **[TIME_WINDOW_WIDTH].[SLICE].urls.changed** *(Integer)*: The number of times the URL changes in the time window
- **[TIME_WINDOW_WIDTH].[SLICE].urls.unique** *(Integer)*: The number of unique website URLs in the time window
- **[TIME_WINDOW_WIDTH].[SLICE].speed.total.{sum, avg, std}** *(Float)*: The total and average (with standard deviation) speed of the mouse in the time window
- **[TIME_WINDOW_WIDTH].[SLICE].speed.x.{sum, avg, std}** *(Float)*: The total and average (with standard deviation) horizontal component of the speed of the mouse in the time window
- **[TIME_WINDOW_WIDTH].[SLICE].speed.y.{sum, avg, std}** *(Float)*: The total and average (with standard deviation) vertical component of the speed of the mouse in the time window

## Technical Validation

The dataset is designed to collect “in the wild” raw interaction data, reflecting real user behaviour in their natural browsing environments. As such, the presence of noise and outliers is expected and considered a natural aspect of user interactions. Unlike controlled laboratory settings, real-world usage is subject to various factors that can introduce variability, including user distractions and varying browsing contexts and goals^20^. Thus, no specific measures were implemented to flag or filter out these irregularities, as they are integral to understanding authentic user experiences. This approach may enable a more comprehensive analysis of user interactions and emotional responses, capturing the complexity of realistic online behaviour rather than idealized scenarios.

To minimize bias in data collection through convenience sampling, recruited participants have various backgrounds and employments, thus naturally varying their browsing behaviour. A comprehensive tracking system was implemented to monitor participants’ interactions, maintaining an accurate log of session details for each user and creating the dataset. Regarding the assessment of the emotional analysis tool, the existing literature underlines that this solution represents the state of the art^12,17^.

### Limitations

We acknowledge several limitations in our study, particularly in relation to data quality and sample size. First, regarding the quality of facial images used for emotion recognition, we clarify that participants were reminded of their involvement in the study upon opening the browser. However, no additional measures were taken to ensure consistent image quality (e.g., evaluation from psychology professionals). To protect participants’ privacy, images were never stored, which also meant that researchers could not retrospectively review them to assess or improve quality. Nonetheless, the AFFDEX SDK includes internal safeguards: poor conditions, such as occluded faces, off-screen gazes, or other distractions, typically result in low engagement scores or, in more severe cases, in a failure to detect a face altogether, which leads to no emotion data being recorded. Still, it must be highlighted that the adoption of the AFFDEX SDK brought some issues. In particular, out of 10,264,985 records that *should* contain emotion data, only in 3,741,316 cases a face was recognized, and among these, only 590,792 (approximately 5.75% of the records that should contain emotion data) *actually* contain some emotion data. This signals that possibly the AFFDEX SDK could not detect a face or did not recognize any emotion (for example, because the user did not correctly set up its webcam, the room was too dark, or due to other suboptimal scenarios). Although a revision of the employed emotion detection and overall pipeline might improve the yield of emotion data (e.g., by allowing manual checks of the webcam snapshots), we rely on the assumption, supported by prior work^21–23^, that with sufficient in-the-wild data, the impact of occasional low-quality inputs would be mitigated through scale.

Second, we acknowledge that the sample size of 19 participants is relatively limited. We addressed this constraint by extending the data collection period, thereby increasing the temporal coverage. Nevertheless, we recognize that broader generalization would benefit from a larger and more diverse sample, since all our participants were Caucasian. We anticipate that, in the future, the dataset may be expanded by adopting the same protocol.

## Data Availability

The dataset is available on FigShare at 10.6084/m9.figshare.28489601.

## Code availability

The data collection process utilized three main components: a custom browser extension to track user interactions, a web server to manage and process the collected data, and an emotion recognition module powered by Affectiva’s AFFDEX SDK. It must be highlighted that we chose to provide sum, average, and standard deviation since prior machine learning tasks seem to adopt these features (see, for example, the following ref. ^1^). However, the dataset can be expanded with additional task-specific features by leveraging the source code we used to compute these basic features. The source code for all components is publicly available on GitHub at https://github.com/uxsad/dataset-tools.
